# The Positive Relationships Between Paranoia, Perceptions of Workplace Bullying, and Intentions of Workplace Deviance in United Kingdom and French Teachers: Cross-Cultural Aspects

**DOI:** 10.3389/fpsyt.2020.00203

**Published:** 2020-03-17

**Authors:** Bárbara Cristina Da Silva Lopes, Catherine Bortolon, Válerie Macioce, Stéphane Raffard

**Affiliations:** ^1^ University of Coimbra, Faculdade de Psicologia e de Ciências da Educação, Coimbra, Portugal; ^2^ Center for Research in Neuropsychology and Cognitive and Behavioral Intervention (CINEICC), Coimbra, Portugal; ^3^ Laboratoire Inter-universitaire de Psychologie Personnalité, Cognition, Changement Social, Université Grenoble Alpes, Grenoble, France; ^4^ Centre Hospitalier Universitaire de Montpellier, Montpellier, France; ^5^ Unité de recherche clinique et epidémiologique, Centre Hospitalier Universitaire de Montpellier, Montpellier, France; ^6^ University Paul Valéry Montpellier 3, University of Montpellier, Epsylon EA, Montpellier, France; ^7^ Service Universitaire de Psychiatrie Adulte, Centre Hospitalier Universitaire (CHU) Montpellier, Montpellier, France

**Keywords:** paranoia, workplace bullying, workplace deviance, negative affect, cross-cultural

## Abstract

Cognitive models of psychopathology were applied to inform the relationships between paranoid cognitions, perceptions of workplace bullying, and intentions of workplace deviance in UK and French teachers. Sixty-six UK teachers and 50 French teachers were asked to fill in an online survey comprised of the Green Paranoia Thought Scales, Negative Acts Questionnaire, Depression, Anxiety and Stress scales, and Workplace Deviance Scale. The variables in this study were conceptualized as cognitions and not as facts because the study used self-report questionnaires of paranoid ideation, workplace bullying, and workplace deviance. Mann-Whitney tests showed that UK teachers report significantly more perceptions of work-related bullying and intentions of workplace deviance than French teachers. However, there was no statistically significant difference between UK and French teachers for the report of paranoid ideation. Mediation analyses showed that paranoia impacted on intentions of workplace deviance but perceptions of workplace bullying and negative affect did not mediate this association in UK and French teachers. Culturally tailored psycho-social interventions should be implemented targeting teachers' paranoid thinking and workplace bullying in order to deter teachers from engaging in workplace deviance and to promote their well-being.

## Introduction

Research on applying clinical cognitive and behavioral models (1, 2) to organizational contexts to explain the relationships between paranoid thinking, perceptions of workplace bullying, and intentions of workplace deviance is in its infancy. A recent cognitive and behavioral model developed by Chan and McAllister (1) proposes to couple organizational theory with clinical theory to explain how paranoid cognitions (i.e., thoughts about the intentionality, malevolence, and persecution of others in doing harm against oneself) (3) and workplace bullying and workplace deviance may be interrelated. Recent studies with UK and French samples of workers show that paranoid thoughts are common in the workplace in both UK and French contexts and that those thoughts are then related to workers' own perceptions of workplace bullying (i.e., being subjected to harassment, intimidation, and acts negatively affecting one's work) (4) and of workplace deviance (i.e., voluntary behaviors that violate the organizations' norms and breach the assigned duties of the job role, e.g., stealing, spreading rumours, consistently arriving late to work or class, etc.) (5) as retaliation against the perceived harm/bullying/persecution of others against the workers (6–8). Moreover, there is an increasing recognition in clinical research that workplace conditions, such as, a threatening workplace environment characterized by the bullying of the employees constitutes a high risk factor for poor mental health in employees (7, 8) and that certain professions may be more at risk of developing specific psychiatric conditions and symptoms e.g., paranoid personality disorder, depression, and depressive symptoms and paranoia, than others (7).

Although some effort has been done in the past to study the prevalence of paranoia and its associations with both perceptions of workplace bullying and workplace deviance in samples of workers mostly from Anglo-saxonic countries, e.g., the UK (7, 8), there is still a lack of studies that focus on specific professions, like teaching, and have cross-cultural samples, which will be addressed in this study. Added to this, most studies in the literature on workplace bullying and on workplace deviance focusing on teachers only have been conducted with samples of teachers from Eastern Europe and Turkey (9–12) and not with samples of countries from central and northern Europe, e.g., France and the UK. This is important to research because those two countries are considered to be the richest countries in Europe, with their citizens having a better quality of life and working conditions compared to other countries in Europe (13).

**Figure 1:**
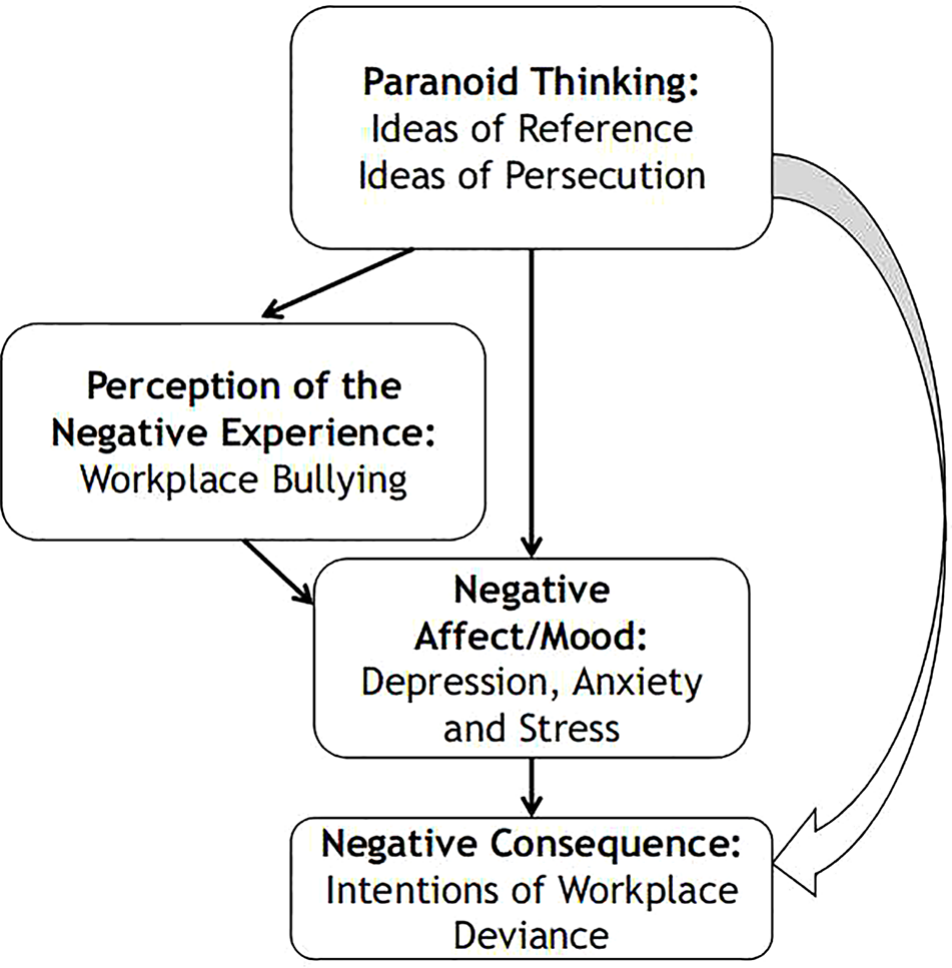
Cognitive model depicting the relationships between paranoia, perceptions of workplace bullying, negative affect, and intentions of workplace deviance in UK and French teachers.

This study will address those issues and will use a cognitive and behavioral framework of cognitions, affect, and behavior (1, 2) and apply it to the workplace by developing a model and applying it to cross-cultural samples of French and UK teachers (see **Figure 1**) and making as well comparisons between the two. This constitutes an advancement to the current literature, because research that was done previously can be considered to be rather a-theoretical and lacking a clear theoretical explanation for the relationships that are observed between workplace bullying and deviance and the poor mental health of teachers (12, 14). Research in this area also uses self-report instruments to measure concepts such as, workplace bullying (4), and those instruments measure perceptions and not actual observable behavior, hence a cognitive perspective should be used to inform relationships between concepts. In conclusion, marrying clinical and organizational theories will constitute an advancement to current theories of mental health in the workplace, and this will be one of the first studies to explore within a cognitive framework the relationships between paranoia, workplace bullying, and workplace deviance in teachers.

### The Cognitive Information-Processing Model for the Relationships Between Paranoia, Workplace Bullying and Negative Behavior in the Workplace

Workplace bullying is defined by Einarsen, Hoel, Zapf, and Cooper [(15), page 11] as “harassing, offending, socially excluding someone or negatively affecting someone's work tasks. In order for the label bullying (or mobbing) to be applied to a particular activity, interaction or process, the bullying behavior has to occur repeatedly and regularly (e.g., weekly) and over a period of time (e.g. about 6 months) (15).” The results of the fifth European Working Conditions Survey, carried out in 2011–2012, suggested that the education sector is one of the sectors that tends to have the highest levels of incidence of workplace bullying (16). Therefore, researching the prevalence of perceived workplace bullying with cross-cultural samples of teachers is quite important.

Previous research has found that more than 20% of both UK and French teachers have reported having received threats of physical violence. In France, more than 10% of teachers reported physical violence from non-colleagues. In the UK, prevalence rates for reported workplace bullying of teachers were higher than 10% and 15%, and the most common types of bullying were physical violence from colleagues and general bullying, respectively (17).

In France, a study by Gilbert and Vercambre-Jacquot (2016) showed that the perpetrator(s) of the bullying of French teachers may depend on the students' teaching grades and social background. In basic education (e.g., nursery and primary school), perpetrators are most often the parents, while in secondary school and high school, perpetrators are most often the students themselves (18). Also, French teachers that teach secondary school and students from a socially disadvantaged background are more vulnerable to violence (18). Moreover, the study also found that institutional staff (co-workers, head teachers, administrative staff, etc.) account for a large share of perpetrators of violence in all levels of education, with a growing share from 27% in kindergarten to 90% in superior levels (18). This study clearly shows that teachers in France are vulnerable to bullying from the parents, students, colleagues, and their superiors. In Great Britain, the Association of Teachers and Lecturers (ATL) reported that 76% of the respondents mentioned that they have been recipients of bullying by their superiors, and 22% by their colleagues. 25% mentioned that they have been bullied by a student and 23% by a parent or guardian (19). Research thus suggested that both French and UK teachers suffer from moderate to high workplace bullying and that workplace bullying of European teachers has very negative effects on them because it has been found to be positively correlated with stress and depression and negatively correlated to self-esteem (9–11, 20).

Workplace bullying has also been found not only to be linked to teachers' poor mental health (20) but also to teachers' workplace incivility/deviant behavior (12). Indeed, workplace deviance of teachers has been found to be a form of retaliation against the school's culture and management style or structure because of teachers feeling injustice, reporting unmanageable workload, lack of support and lacking promotion opportunities, or because teachers suffer from bullying perpetrated by school headmasters, other colleagues and students (12). Nonetheless, it is also commonly accepted that all forms of teachers' deviant behaviors, whether overt or covert, are harmful for the school and students (21).

Indeed, teachers' workplace deviant behaviors may affect the students' learning progression and well-being, and the quality of teachers' relationships with other colleagues and with the school management, impacting negatively, as well on the well-being of teachers. Therefore, detection of deviant behaviors in schools is critical to prevent such behaviors and to take necessary counter-measures. In spite of this, previous research on this important topic has failed to explain what are the reasons behind the perceptions and report of workplace bullying and of workplace deviance by teachers. This study will aim to address this lacuna by drawing on cognitive models of psychopathology and bullying in the workplace.

According to classic cognitive and behavioral models of psychopathology (2, 22) that take an information processing approach, a trait that represents vulnerability toward psychopathology (e.g., a paranoid trait) is composed of specific schemata e.g., paranoid schemata that show certain types of thoughts or beliefs, in this case, paranoid thoughts. These paranoid schemata then filter information from the social world and skew information processing. These traits that constitute vulnerability to psychopathology are also shaped by experiences and influence the perception of experiences, in this case, the experience of workplace bullying, to provoke specific maladaptive cognitions (e.g., intentions of workplace deviance). Hence, whenever negative events happen to the worker, they activate particular schemata that will influence and skew the processing of information from the social environment, producing negative cognitions that will help to guide behavior (1).

As such, we would expect under the cognitive and behavioral models of psychopathology and bullying (1, 2, 22) that the report of workplace bullying by the employees is done through the lens of their paranoid thinking that is characterized by heightened hypervigilance toward threat-related information (e.g., possible sources of abuse and of bullying in the workplace), rumination, as well as elevated levels of stress, depression, anxiety and fear. Therefore, it is assumed that because employees are paranoid, they would also perceive workplace bullying and vice-versa and that paranoia and perceptions of workplace bullying may induce negative affect/mood (i.e. depression, anxiety, stress), which in their turn may trigger and maintain the paranoia (2). Hence, a vicious circle may be installed between paranoia, perceptions of workplace bullying and negative affect where they feed and maintain each other.

Moreover and according to Chan and McAllister's (1) model of abusive supervision through the lens of paranoia (1), a worker's paranoia may be maintained even after the bullying has ceased to occur because paranoia is associated with information processing errors that (mis)attribute malevolent intentions to other people's behaviors toward the worker in the workplace without evidence supporting this attribution (i.e., the sinister attribution error) (1). Also and under this model, it is assumed that workers will show different safety behaviors that can include avoidance, but also aggression (e.g., workplace deviant behavior) to cope with the paranoid and bullying cognitions and associated negative affect and as retaliation against the perceived abuse and persecution in the workplace (1). In addition, workers' workplace deviant behaviors are likely to induce responses from others in the workplace that are abusive and negative, consequently, those behaviors might help trigger workers' paranoid thoughts about the malevolence of others and might maintain them in the long term (1).

It is possible that in time, the vicious circle between paranoid thinking, perceptions of workplace bullying and negative affect/mood and intentions of workplace deviance may predispose the onset of psychiatric disorders like psychosis and or major depression (7, 23, 24). Therefore, and by following those cognitive and behavioral models, we propose to study the relationships between paranoia, perceptions of workplace bullying, negative affect/mood, and intentions of workplace deviance so that preventive measures can be taken. We also argue that paranoia is a trait schema that is composed of beliefs of a grand conspiracy and of a malicious and intentional threat of others against oneself that are associated with the perception of workplace bullying (1), with negative affect (2) and with workplace deviance (8) in UK and French teachers.

It is important to note that in the model (see **Figure 1**) we devised to explore the relationships between the variables we conceptualise paranoia (cognitions) as having uni-directional and direct and indirect effects on intentions of workplace deviance (cognitions) through perceptions of workplace bullying and or negative affect (depression, stress, anxiety). Although we acknowledge as proposed by Chan and McAllister (1) that there might be bi-directional relationships between paranoia, negative affect, bullying and workplace deviance (1) where paranoia feeds into them and then those in turn feed the paranoia, we propose to explore how paranoia might in a uni-directional way have direct and indirect effects on intentions of workplace deviance.

This is proposed because first, there is clinical/genetic research on paranoia that has shown that paranoia may not be as influenced by negative experiences as to be expected (e.g., workplace bullying that happens during adult life) and as such, has a strong genetic component (25, 26). Second, recent experimental research showed that one's negative cognitions and behaviors (e.g., prejudice) in response to “salient” people that are perceived as “threatening” (e.g., Muslisms) are not influenced by one's exposure to negative events involving those people (e.g., news about jihadi terrorism) but by one's level of paranoia (27). Added to this, other research has also found that paranoia by itself is capable of influencing both cognitions and behavior (e.g., social cognitions and performance in a task) when controlling for both negative affect and the context where the behavior takes place (28).

In conclusion, there is research that seemed to suggest that on the one hand, paranoia may not be as influenced by negative experiences like workplace bullying as previously thought, and on the other hand, paranoia is capable of influencing other negative context-dependent cognitions and behavior independently of the exposure to context-dependent negative events that are thought to prompt them and of the presence of negative affect. As such, we propose a model that looks specifically at the uni-directional influence of paranoia on intentions of workplace deviance that is either direct or indirect through the influence of perceptions of workplace bullying and or of negative affect.

Also, workplace bullying and negative affect were proposed as possible mediators of the influence of paranoia on intentions of workplace deviance because on the one hand, it was found in samples of UK workers that paranoia is likely to interact with perceptions of abuse in the workplace to explain intentions of workplace deviance that in its turn, works as retaliation against the perceived abuse in the workplace through the lens of paranoia (8). On the other hand, research in France and in contrast to the UK, has suggested that paranoia in the workplace may also be linked to the presence of negative affect (6), so this variable will be included in the analyses within the French sample of teachers.

### Hypotheses

H1. It is expected that UK teachers would show statistically significantly more work-related bullying than French teachers and that they would also show statistically significantly more intentions of workplace deviance than French teachers.

H2. Drawing on the cognitive and behavioral model of abusive supervision through the lens of paranoia (1), we expect to find positive relationships between paranoid thinking and perceptions of bullying and intentions of workplace deviance in both samples of teachers.

H3. Drawing on the previous cognitive and behavioral models and research on the effects of sub-clinical paranoia on cognition and behavior (28), we expect to find direct and indirect effects of paranoid thinking (trait-disposition) on intentions of workplace deviance (consequence). It is expected that paranoia has direct and indirect effects on intentions of workplace deviance for both UK and French samples of teachers. For the UK sample, it is expected that paranoid thinking has a direct effect on intentions of workplace deviance but also that workplace bullying acts as a mediator for the effect of paranoid thinking on intentions of workplace deviance. Similarly, for the French sample, it is expected that paranoid thinking has an indirect effect on intentions of workplace deviance when both workplace bullying and negative affect/mood are inserted in the model as mediators.

## Materials and Methods

### Participants and Procedure

In total, 50 French and 66 UK teachers were included in the present study. The inclusion criteria were to be currently working as a teacher for the past 6 months and teaching different teaching grades ranging from elementary to high school levels excluding university. Teachers that had managerial and supervisory roles were excluded from the analyses. The recruitment of teachers in the UK was made by contacting the headmasters of public schools and specialized schools in Nottingham and then asking for permission to advertise the study to teachers by distributing fliers with the link to the online qualtrics survey hosted at Nottingham Trent University in the UK and by advertising the study in the schools' webpage dedicated to teachers only. Teachers were told in both samples that the study was about negative experiences at school and workplace performance of teachers to avoid socially desirable responses.

Regarding the French sample, recruitment of participants was made through four methods: by sending an invitation email to participate in this study through social networks, especially groups and associations of teachers; through the websites of teacher associations (APMEP Association of Teachers of Mathematics of Public Education, AEFVDA Teachers' Association of Valle d'Aosta, APHG Association of History-Geography Teachers) among others and through contacts by telephone and email to the secretariats of some French educational institutions. Acquaintances were also requested to diffuse the invitation email to their colleagues working in schools in France. Participants in the French study completed an online survey hosted at Epsyline, the online questionnaire service of the Epsylon Research Laboratory. In both studies, the first page of the survey contained a brief introduction to the study, followed by socio-demographic questions. Those questions were then followed by mental health questions that then were followed by the battery of the questionnaires used in this study. No identifying data was requested in both UK and French studies and both online surveys asked for teachers' consent by asking them to click “yes” to continue to the online survey. Moreover, participants in both studies were allowed to drop out at any time by just closing the web browser. They were also fully debriefed at the end and the study took approximately 30 minutes to complete. All data were anonymized by the qualtrics and or Epsylon software that generated a random number for the participants' data. The studies also followed the British Psychological Society's code of ethics for internet research and the code of ethics of the World Medical Association (Declaration of Helsinki) and were approved by the Ethics Research Committees of Nottingham Trent University's Department of Psychology in the UK and the Laboratory of Epsylon in France.

### Questionnaires

Paranoia was measured by the **Green Paranoia Thoughts Scale** (**GPTS**) (29). The GPTS is a self-administered tool that measures both ideas of social reference and persecutory ideas that compose a paranoid trait schema. The GPTS is composed of a total of 32 items rated on a 5-point Likert scale from 1 (Not at all) to 5 (Totally). Items are grouped into two 16-item scales. Scale A assesses ideas of social reference relevant to paranoia, while scale B assesses persecutory thoughts. Scores in each scale range from 16 to 80 points, with higher scores reflecting a higher level of paranoid thinking and a tendency to be paranoid. The French version of this scale was translated using back-translations according to Sousa and Rojjanasrirat (30). The original authors of the GPTS report high reliability for this scale with Cronbach alphas of .95 and .90 (29). In this study, this scale also shows good reliability for both the UK sample (GPTS Ideas of reference: α = 0.98 and GPTS Ideas of persecution: α = 0.98) and for the French sample (GPTS Ideas of reference: α = 0.90 and GPTS Ideas of persecution: α = 0.96).

The perception of victimisation by behaviors constituting bullying/harassment in the workplace, as well as the frequency of the occurrence of these behaviors in the last 6 months were measured by the **Negative Acts Questionnaire-Revised (NAQ)** (4) which includes 22 items, without directly using the term “psychological harassment”, on a five-point scale ranging from (1) Never to (5) Daily. Both the UK and French samples showed very good internal consistency for the NAQ in this study (total sample NAQ: α = 0.94; for the the UK sample: α = 0.96; for the French sample: α = 0.86). The items of the questionnaire are divided into three distinct factors: Work-related Bullying, Personal-related Bullying, and Physically intimidating bullying. The French version of this questionnaire was translated and validated by Fournier (31).

The intentions to engage in deviant behaviors that violate organizational norms and consequently the well-being of the organization and of its members were measured by the **Workplace Deviance Scale (WD)** (5). This scale asks participants to evaluate the frequency in which they intend to violate the organization norms/rules after thinking about being victims of workplace bullying in the past 6 months. The scale is composed by 28 items evaluating both organizational deviance (deviant behaviors directly harmful to the organization) and interpersonal deviance (deviant behaviors directly harmful to other individuals within the organization). The French version was translated using back-translations according to Sousa and Rojjanasrirat (30). Consistent with the Cronbach alphas reported by the original authors of the scale (5) the scale shows excellent internal consistency for the two samples of this study (UK sample: α = 0.96, French sample: α = 0.83).

The **Depression, Anxiety and Stress Scale (DASS-21)** was also employed to measure negative affect and mood including depression, anxiety, and stress in the French sample. Each item is scored on a 4-point scale (never—all the time). Higher scores indicate greater levels of negative affect and mood. It was initially developed and validated by Parkitny and McAuley (32). The French version of this scale was validated by Ramasawmy (33), suggesting a good internal reliability (Cronbach alphas range from 0.72 to 0.79). In this study, the French sample's Cronbach's alpha for this scale indicated an excellent internal reliability (α = 0.92).

### Statistical Analysis 

Data analyses were performed using SPSS software (version 20). Exploratory data analyses revealed that the data distributions were not normal. We tried to deal with the non-normal distributions by using transformations. The methods employed did not result in normal data distributions. Consequently, we chose to perform non-parametric analyses. Comparisons between the two samples (UK vs. French samples of teachers) were performed using Mann-Whitney analyses. Moreover, correlations between the different variables employed in this study were analysed using Spearman's Rho correlations for each sample. Benjamini and Hochberg's (34) false discovery rate (FDR) (34) and corrected significance level were applied to the comparisons and correlations to reduce the risk of false positives and of Type I errors. Indeed, Benjamin and Yekutieli (35) argue that the FDR may be the appropriate error rate to control in many applied test problems and was shown to be more poweful than comparable procedures that control the traditional familywise error rate (35).

A power analysis performed with G* power package was conducted to examine the strength of the differences between the uneven groups. It was observed for a sample of 116 individuals a power sensitivity of 99% for the two samples' comparisons for work-related bullying and workplace deviance and its dimensions, which enabled us to reject the null hypothesis and hence to support hypothesis 1. We used the SPSS macro developed by Hayes (36) to test our mediation models for the UK and French samples of teachers. In this study, a bootstrapping procedure based on 5,000 resamples was used to calculate a 95% bias-corrected confidence interval (BCCI) around the total indirect effect. Both regression and mediation analyses do not request data to be normally distributed but request errors to be normally distributed. Besides, assumptions for these analyses also include linearity (Normal Probability Plot), homoscedasticity (Plot of residuals versus predicted value), independence (Durbin-Watson statistic) of Residuals, the presence of outliers (Cook's distance < 1) and multicollinearity (VIF < 2) (36). These assumptions were all tested and no major problems were found, except for a problem with homoscedasticity. Transformations were applied to correct this issue.

## Results

### Socio-Demographic Characteristics and Mental Health Disorders

The samples' characteristics concerning the socio-demographic variables and the report of mental disorders are presented in **Table 1**. There were no statistically significant differences between the two samples of UK and French teachers regarding the proportion of male/female participants, nor regarding the proportion of participants currently receiving psychiatry treatment and currently suffering from a psychiatric disorder. It important to note that regarding the prevalence of mental disorders, although there were no significant differences between UK and French teachers for the report of psychiatric diagnoses, more UK teachers seem to report psychiatric disorders than the French and more UK teachers seem to be currently receiving psychiatric treatment than the French (6% vs. 3% for psychiatric disorders and 5% vs. 2% for psychiatric treatment, respectively). In addition to this, the most common psychiatric disorder reported by UK teachers was depression.

**Table 1:** Socio-demographic, clinical, and work-related variables.

	UK Sample *N* = 66		French Sample *N* = 50		
	*N*	%	*N*	%	Statistic
**Gender (males)**	12	7.92	8	4.00	χ2 = 1.399, *p* = .49
**Gender (females)**	54	35.64	42	84	
**Currently receiving Psychiatric treatment**	8	5.28	3	1.50	χ2 = .892, *p* = .35
**Received a Psychiatric Diagnosis**	10	6.60	5	2.50	χ2 = .607, *p* = .44
*Anxiety*	1	.66	2	1.00	
*Depression*	5	3.30	1	.50	
*PTSD*	1	.66		.00	
*Work related stress*	1	.66		.00	
*Burn-out*		.00	1	.50	
*Psychosis*		.00	1	.50	
*Stress/Anxiety/Depression*	1	.66		.00	
**Job Role**					
*Teacher*	49	74.2	42	84	
*Specialized Teacher*	13	19.7	4	8	
*Assistant Teacher*	4	6.1			
*Not known*			4	8	
**Level Taught**					
*Not known*	2	3	3	6	
*All levels*	4	6.1	3	6	
*Nursery*	1	1.5			
*Primary School*	27	40.9	15	30	
*Secondary School*	32	48.5	18	36	
*High School*			11	22	

### Total Sample Characteristics and Comparisons Between UK and French Samples

The means and sds for the key variables for the total sample of teachers (*N* = 116) are presented in **Table 2**.

**Table 2:** Means and Sds for the key variables.

	*Mean*	*SD*	*Min*	*Max*
**Green Paranoia Thoughts Scale (GPTS)**				
*Ideas of Reference*	30.16	15.63	16	80
*Ideas of Persecution*	25.97	15.98	16	79
**Negative Acts Questionnaire (NAQ)**				
*Work-Related Bullying*	12.91	5.47	7	29
*Personal-Related Bullying*	17.11	7.49	12	45
*Physically intimidating*				
*bullying*	3.96	1.80	3	13
*Total Score*	33.97	13.37	22	80
**Workplace deviance (WD)**				
*Interpersonal Deviance*	11.58	5.26	7	36
*Organizational Deviance*	20.63	9.75	12	59
*Total score*	45.76	19.97	27	141

Subsequently, **Table 3** presents the means and standard deviations for each of the continuous variables separately for each sample.

**Table 3:** Comparisons between the UK and French samples of Teachers for the key variables.

	UK Sample (*N*=66)		French Sample (*N*=50)		*Statistic*
	*Mean*	*SD*	*Mean*	*SD*	
**Age**	36.86	9.22	38.24	11.56	*U* = 1598.5, *p* = .77
**Years of Teaching Experience**	11.41	7.97	12.54	10.86	*U* = 1622.0, *p* = .88
**Green Paranoia Thoughts Scale (GPTS)**					
*Ideas of Reference*	29.24	16.51	31.36	14.47	*U* = 1360.5, *p* = .11
*Ideas of Persecution*	25.56	14.64	26.52	17.74	*U* = 1628.0, *p* = .90
*Total Score*	54.80	30.60	57.88	29.60	*U* = 1408.0, *p* = .18
**Negative Acts Questionnaire (NAQ)**					
*Work-Related Bullying*	14.24	6.16	11.14	3.77	*U* = 1173.0, *p* = .008**
*Personal-Related Bullying*	18.17	8.90	15.72	4.82	*U* = 1504.5, *p* = .41
*Physically intimidating*					
*Bullying*	4.06	2.05	3.82	1.41	*U* = 1643.0, *p* = .96
*Total Score*	36.47	15.89	30.68	8.06	*U* = 1378.0, *p* = .13
**Workplace deviance (WD)**					
*Interpersonal Deviance*	12.54	6.13	10.32	3.50	*U* = 1291.0, *p* = .044*+
*Organizational Deviance*	23.07	11.68	17.42	4.86	*U* = 1152.0, *p* = .005**
*Total score*	50.67	24.10	39.28	9.47	*U* = 1181.5, *p* = .009**

*p ≤ .05, **p ≤ .005.

Benjamini and Hochberg's (34) corrected significance level q = 0.015.

+Not statistically significant when Benjamini and Hochberg's (34) corrected significance level is used.

#### Acknowledgment of Workplace Bullying in the Past 6 Months by UK and French Teachers

Concerning the report of workplace bullying in the past 6 months (item 23 of the NAQ) (4) 68% of UK teachers did not acknowledge suffering from workplace bullying in the past 6 months, whereas 17% acknowledged suffering from workplace bullying in the past 6 months but rarely and 11% acknowledged workplace bullying now and then and 5% acknowledged suffering from bullying several times a week. In contrast to this, more French (80%) than UK teachers did not acknowledge suffering from workplace bullying in the past 6 months, whereas 16% acknowledged suffering bullying in the past 6 months but rarely and only 2% acknowledged suffering from workplace bullying several times a week and 2% daily. Also, the majority of the UK teachers perceived their immediate superiors to be the perpetrators of the workplace bullying (*N* = 19; 73%). In contrast to this, the French teachers identified evenly the perpetrators of workplace bullying as being their immediate superiors (*N* = 3), colleagues (*N* = 3), and students (*N* = 3).

#### Differences Between UK and French Teachers for the Variables of Interest

Mann-Whitney tests showed statistically significant differences between UK and French samples for work-related bullying and for the two sub-scales of workplace deviance (organizational and interpersonal deviance), as well as for the workplace deviance score. After applying Benjamini and Hochberg's (34) corrected significance level (*q* = 0.015), UK teachers showed statistically significantly more work-related bullying and more intentions of workplace deviance and more specifically, organizational deviance than French teachers (see **Table 3**). In contrast to this, no statistically significant differences were found between French and UK teachers for their levels of paranoid thoughts (both ideas of reference and persecution).

#### Prevalence of Paranoid Thoughts in UK and French Samples of Teachers

Echoing previous findings with an UK non-clinical population (29), the means for the Ideas of Social Reference (more common paranoid ideas- hierarchy of paranoid thoughts) (3) are higher than the means for the Ideas of Persecution of the GPTS (29) both for UK and French teachers (*M* = 29.24, *SD* = 16.51 for the UK's and *M* = 31.36, *SD* = 14.47 for the French's ideas of reference and *M* = 25.56, *SD* = 14.64 for the UK's and *M* = 26.52, *SD* = 17.74 for the French's ideas of persecution of paranoia). Concerning the prevalence of paranoid thoughts of the GPTS, ranging from the more common thoughts concerning ideas of reference to ideas of persecution of different levels of threat including a conspiracy, UK teachers showed a *M* = 1.71, *SD* = 1.26 for the thought “People definitely laughed at me behind my back” and 12% of UK teachers fully agree that they thought that people laughed behind their back. Similarly to the UK teachers, French teachers showed a *M* = 1.76, *SD* = 1.38 for “People definitely laughed at me behind my back” and 10% of French teachers acknowledged completely having this thought. Relatively to the more clinical thoughts of paranoia, UK teachers showed a *M* = 1.72, *SD* = 1.25 for the thought “I have definitely been persecuted” whereas French teachers showed a slightly lower mean for this thought (*M* = 1.58, *SD* = 1.37). This meant that both UK and French teachers tend to somewhat agree to having the thought of being persecuted. Moreover, 8% of UK teachers and 12% of French teachers completely acknowledged being persecuted. Finally, concerning the more severe thought of a conspiracy, UK teachers showed a *M* = 1.53, *SD* = 1.07 and French teachers showed a *M* = 1.40, *SD* = 1.42 for the thought “I was convinced there was a conspiracy against me”, suggesting as expected, that thoughts of a conspiracy are less endorsed by individuals. Moreover, only 5% of UK teachers and 8% of French teachers fully acknowledge having the thought of a conspiracy against them.

### Correlations Between Variables in UK and French Samples

Spearman Rho's correlation coefficients are presented in **Table 4** for UK teachers and **Table 5** for French teachers. Benjamini and Hochberg's (34) corrected significance level (*q* = 0.023) was applied for the French sample of teachers only because *p*-values for the UK sample of teachers were all < 0.0001, therefore no corrections were applied for the correlations with this sample. Additional correlations were performed between DASS-21 negative affect/mood dimensions (stress, anxiety, and depression) and the other continuous variables for the French sample only. DASS -21 depression was significantly positively associated with both paranoia (r_s_ = .29, *p* = .039), and intentions of workplace deviance (r_s_ = .31, *p* = .030). DASS- 21 depression was also moderately positively associated with the physical intimidation dimension of workplace bullying (r_s_ = .28, *p* = .030). DASS-21 anxiety was significantly positively associated with paranoia (r_s_ = .35, *p* = .014) and DASS-21 stress was significantly positively associated with intentions of workplace deviance (r_s_ = .36, *p* = .012) Finally, the DASS-21 total score was significantly positively associated with paranoia (r_s_ = .31, *p* = .026) and with intentions of workplace deviance (r_s_ = .33, *p* =.021).

**Table 4:** Spearman Rho's correlations between the key variables for the UK sample of Teachers.

	UK Sample						
	Age	GPTS Ideas of Reference	GPTS Ideas of Persecution	GPTS total	Workplace deviance total	WD Interpersonal Deviance	WD Organizational Deviance
GPTS Ideas of Reference	-.03						
GPTS Ideas of Persecution	-.09						
GPTS Total score	-.05						
Work-Related Bullying	-.04	.69^**^	.54^**^	.66**	.48^**^	.49**	.51**
Personal-Related Bullying	-.01	.75^**^	.72^**^	.76**	.65^**^	.61**	.61**
Physically intimidating bullying	-.16	.55^**^	.62^**^	.59**	.48^**^	.48**	.47**
Negative Acts Questionnaire Total score	-.05	.77^**^	.66^**^	.76**	.56^**^	.57**	.59**
WD Workplace deviance Total score	-.02	.68^**^	.72^**^	.71**			
WD Interpersonal Deviance	-.06	.57**	.62**	.59**			
WD Organizational Deviance	-.03	.69**	.73**	.72**			

All p-values < 0.0001; ** p < .001 no correction was applied.

**Table 5:** Spearman Rho's correlations between the key variables for the French sample of Teachers.

	French Sample						
	Age	GPTS Ideas of Reference	GPTS Ideas of Persecution	GPTS total	Workplace deviance total	WD Interpersonal Deviance	WD Organizational Deviance
GPTS Ideas of Reference	-.05						
GPTS Ideas of Persecution	-.02						
GPTS Total score	-.09						
Work-Related Bullying	.03	.35*	.32	.33*	.12	.07	.15
Personal-Related Bullying	.18	.44*	.56*	.51*	.30	.28	.24
Physically intimidating bullying	.25	.24	.45*	.37*	.17	.25	.11
Negative Acts Questionnaire Total score	.11	.50*	.57*	.54*	.26	.24	.22
WD Workplace deviance Total score	.00	.38*	.27	.39*			
WD Interpersonal Deviance	.19	.24	.19	.26			
WD Organizational Deviance	-.13	.36*	.22	.36*			

*Benjamini and Hochberg's (34) corrected significance level q = 0.023; * p < .023.

### Mediation Models for the UK and French Samples of Teachers 

Following the results of correlations for both samples, one mediation model was tested for the UK sample using intentions of workplace deviance as a dependent variable, paranoid thinking measured by the GTPS (29) as an independent variable and workplace bullying measured by the NAQ (4) as a mediation variable. Results showed that paranoid thinking was significantly associated with both workplace bullying (path a; *β* = .45, *p* = .001; *R* ^2^ = .76, *p* = .001) and with intentions of workplace deviance (path c; *β* = .41, *p* = .001), nevertheless, workplace bullying was not statistically associated with intentions of workplace deviance (path b; *β* = .43, *p* = .08; *R* ^2^ = .61, *p* = .001). Consequently, the indirect effect of paranoid thinking on intentions of workplace deviance through workplace bullying was not statistically significant (*β* = .19, *SE* = .16, BCBCI 95% .096–.53).

Regarding the French sample, paranoid thinking was entered as an independent variable and intentions of workplace deviance as a dependent variable. Since workplace bullying was neither associated with negative affect/mood nor with intentions of workplace deviance, we could not enter this variable as a mediator in the model as we did for the UK sample. Instead, we entered the variable of negative affect/mood as a mediator since correlations showed that this variable was associated with intentions of workplace deviance in the French sample of teachers. Therefore, we decided to move forward with the testing of the mediation effect of negative affect/mood. Results showed that paranoid thinking was statistically significantly associated with negative affect/mood (path a; *β* = .09, *p* = .04; *R* ^2^ = .08, *p* = .04). Nevertheless, neither negative affect/mood (*β* = .26, *p* = .72) nor paranoid thinking (*β* = .08, *p* = .67) were significantly associated with intentions of workplace deviance (*R* ^2^ = .41, *p* = .10). Consequently, the indirect effect of paranoid thinking through negative affect/mood on intentions of workplace deviance although being statistically significant (37), it cannot be considered. (*β* = .02, *SE* = .015, BCBCI 95% .001–.06).

## Discussion

The study used a cognitive framework to understand the relationships between paranoia, perceptions of workplace bullying, negative affect and intentions of workplace deviance with samples of UK and French teachers, examining as well differences between the two samples for those variables. First, Mann-Whitney results suggested that UK teachers showed statistically significantly more work-related bullying than French teachers, thus supporting hypothesis 1. Also, looking at their report of bullying in the past 6 months in the NAQ (4), more UK teachers report occasional bullying than French teachers (28% UK teachers vs. 16% French teachers) and UK teachers consistently report their immediate superiors as being the bullies, whereas French teachers report different types of bullies including immediate superiors, colleagues and students. Those results suggested that UK teachers seemed to show more workplace bullying than French teachers and that they perceived their bullies to be their superiors, while French teachers perceived their bullies to be different people. Results thus supported previous research that suggested that workplace bulling of UK teachers is moderately high and that the majority of the perceived bullies of UK teachers are the teachers' superiors (19). Moreover, although French teachers also report being victims of workplace bullying they tend to report less work-related bullying than UK teachers and also tend to report different types of bullies (18). Those results also reflect cultural differences regarding the structure of the educational systems in the UK and in France. Indeed, in France, secondary school teachers do not have a direct supervisor. The National Education Inspectors, who are the direct supervisors of teachers, oversee different educational institutions within a region. Consequently, they are not in direct contact with teachers on a daily basis. Theoretically, the school director and the principal don't occupy a hierarchical position over the teachers in the French education system. Therefore, it is not surprising that French teachers in contrast to UK teachers, report bullying also by their colleagues and students.

Second, paranoid thoughts of the GPTS (29) are relatively common for both UK and French teachers and *on average* both French and UK teachers acknowledge having paranoid thoughts, ranging from ideas of reference to the less common ideas of a conspiracy. Nevertheless, there were no statistically significant differences between French and UK teachers for their levels of paranoid thoughts (both ideas of reference and persecution). However. the means for ideas of reference and of persecution of both UK and French teachers are slightly higher than the means observed in the UK non-clinical sample but not the clinical sample in the original study that validated the GPTS (29) (*M* = 27 vs. *M* = 29 for UK teachers and *M* = 31 for French teachers for ideas of reference and *M* = 22 vs. *M* = 26 for UK teachers, and *M* = 27 for French teachers for ideas of persecution, respectively). Those results suggest that both UK and French teachers tend to have paranoid thoughts and as such, those thoughts should be assessed more deeply in the future with larger national samples.

Results also showed a statistically significant difference between UK and French teachers for intentions of workplace deviance, specifically for the organizational deviance type and this result also supports hypothesis 1. This seemed to suggest in accordance to past literature that workplace bullying is related to workplace deviance in teachers (12). Therefore, because UK teachers showed more workplace bullying, they would also show more intentions to engage in deviant behaviors compared to French teachers and vice-versa.

It is important to note as well that looking at the difference between UK and French teachers for the report of psychiatric diagnoses, although not significant, 6% of UK teachers reported having a psychiatric disorder compared to only 3% of French teachers and that the most common mental disorder for UK teachers was depression, while French teachers reported different disorders but more anxiety. Those results are in agreement to what was found in previous literature on the national prevalence of mental disorders in UK teachers (38, 39) and give support to the notion that French teachers seem to be under-reporting mental issues (40).

Although there is literature to suggest that French teachers are also victims of workplace bullying including physical assault and harassment (17), the reason behind the differences for the report of workplace bullying and intentions to engage in workplace deviance between our UK and French samples of teachers may have to do with the social stigma in France concerning the report of personal mental disorders and of issues in the workplace and of personal unethical behaviors in the workplace (41). Indeed, there is research to suggest that in contrast to UK teachers (39), French teachers tend to under-report issues, not only issues that have to do with their mental health (e.g., burnout and mental disorders) (40, 42, 43) but also the report of personal behaviors that are perceived as being deviant and unethical.

As support for this argument, there was a low level of adherence to the study protocol for the French sample that did not happen with the UK sample of teachers, which may have been explained by the fact that French teachers may have felt ashamed or uncomfortable to report negative experiences in the workplace environment. They may also be feeling even more ashamed to report their own deviant behaviors. Moreover, some French institutions refused to participate in the study due to the kind of questions we were evaluating (e.g., workplace deviance).

Teachers in France besides having the usual teaching responsibilities are also expected to educate students on various social and moral topics such as, on how to behave in a social group and not to steal (44). Therefore, those additional responsibilities of French teachers that include teaching ethical behaviors may prevent them from committing deviant behaviors and also discourage teachers to report them. Also, cultural aspects may impact on the way individuals deal with shame and guilt regarding acts of violence. In fact, UK and French individuals may show different patterns of responses after committing violent acts (e.g., traffic violence) (45), which may be partially explained by the way they deal with feelings of guilt and shame (46). Hence, individuals from the UK may feel less ashamed to report intentions to engage in deviant behaviors when compared to French individuals. Moreover, it seems that in the UK and in contrast to France (see study on the French's public attitudes toward mental health and specifically toward schizophrenia) (41), the stigma of the general public concerning mental illness and the report of workplace issues has decreased and the knowledge of mental disorders has increased in the past years due to the growth of mental health awareness campaigns in the workplace done by charities like Mind (47). Those important cultural differences concerning attitudes toward mental illness and knowledge of mental disorders and regarding the report of intentions to engage in deviant behavior may therefore explain the different results for UK and French teachers.

In addition to those results, our results also supported hypothesis 2 and suggested as previously found in the literature, that paranoid thinking is associated with perceptions of workplace bullying (Lopes' 2013 study with clinical populations) (24) and with intentions of workplace deviance [Lopes et al.'s (8) study with UK workers] and that perceptions of workplace bullying are also associated with intentions of workplace deviance in teachers (12) but for the UK sample of teachers only. The relationship between perceptions of workplace bullying and intentions of workplace deviance in the UK sample may be explained by the fact that when UK teachers feel injustice and are harassed and bullied in the workplace, they may also intend to show workplace deviance as a form of revenge and as a retaliatory strategy to impose equity and fairness, i.e. an eye for an eye, a tooth for a tooth (48) and as a coping response to manage their paranoid beliefs of the perceived malevolence of other people against them in their workplace (8). The fact that perceived workplace bullying did not relate significantly to intentions of workplace deviance in the French sample may be due to the small sample size of the French sample.

In support of Garety, Kuipers, Fowler, Freeman and Bebbington (2) model of persecutory delusions (2), it was observed that paranoid thinking is associated with negative affect (i.e. symptoms of depression, anxiety and stress) in the French sample, which means that paranoia may predispose the onset of negative affet/mood, which in its turn may also predispose the onset of paranoia (3, 23). Moreover, in support of the cognitive model of abusive supervision by Chan and McAllister (1) the perception of bullying is associated with paranoid thinking in both UK and French teachers, meaning that the more teachers perceive workplace bullying, the more they report paranoia and vice-versa. However an in contrast to what was found by Bernotaite and Malinauskiene (14) in their sample of Lithuanian teachers (14), workplace bullying was not statistically significantly associated with negative affect in the sample of French teachers and this may be explained by the fact that the bullying that French teachers experienced was perceived to be rare.

When examining the mediation models for both cross-cultural samples of teachers it was observed in the UK sample that although paranoid thinking had a direct effect on the variance of intentions of workplace deviance, perceptions of workplace bullying (in spite of being associated with intentions of workplace deviance as well) did not act as a mediator for the paranoia's effect on the variance of intentions of workplace deviance. This partially supports our hypothesis 3 that hypothesised direct and indirect effects of paranoia thinking on the variance of intentions of workplace deviance and this result suggested instead that paranoid thinking by itself is sufficient to impact on the variance of intentions of workplace deviance. This result also partially supports the cognitive and behavioral model of paranoia by Garety Kuipers, Fowler, Freeman and Bebbington (2) because it would have been expected under this model that workplace bullying would also have an effect on intentions to engage in negative behavior. Added to this, past research suggested that paranoia is associated with both bullying (24) and with intentions to engage in negative behaviour (8) in the workplace and that experiences of bullying influence the presence of negative behavior in the workplace (12), but our results did not support this.

Nevertheless, the fact that the study found a direct effect of paranoia on the variance of intentions of workplace deviance without workplace bullying for the UK sample of teachers supports the notion in clinical psychology that paranoid tendencies are relatively stable and have a strong genetic component and that they are not easily influenced and shaped by negative events (e.g., workplace bullying) (26). For example, Trower and Chadwick (49) claim that environmental influences are not the root cause of paranoia but rather the genetic make-up of the individual (49). Trower and Chadwick (49) substantiate this claim by pointing out the high rate of hereditary illness in clinical populations, and the 40% concordance rate among monozygotic twins for the presence of paranoia (49). Added to this, support for a strong genetic component for paranoia comes from a recent study that found evidence supporting that a genetic variation in the single nucleotide polymorphism rs850897 is associated with the presence of ideas of reference and paranoia measured by the subscales of the schizophrenia spectrum (25).

Moreover, research has shown the prevalence of a paranoid personality type in non-clinical populations, which might have more explanatory power than environmental factors such as, the experience of bullying (50). In support of this, research by Livesley (26) suggests that some individuals possess certain personality traits which are conducive to paranoid tendencies—such individuals remain in a state of sub-clinical paranoia despite environmental factors (26). Therefore, this clinical literature supports the results for the UK sample of teachers by suggesting that paranoid thinking is a state of a sub-clinical paranoid predisposition that influences behavior and cognitions in spite of environmental factors such as, workplace bullying.

Results of the mediation analysis for the French showed that negative affect did not mediate the impact of paranoia on intentions to engage in workplace deviance, and this also partially supports hypothesis 3. This result meant that perceptions of workplace bullying did not act as a mediator for both UK and French samples. Likewise, negative affect did not mediate the association between paranoid thinking and intentions of workplace deviance in the French sample.

This result may be explained due to the weaker relationships between paranoid thinking and intentions of workplace deviance in the French sample of teachers that may have been caused by the small sample size of the French sample that led to a lack of statistical power in the model. However, this result also fits with the literature on paranoia that argued that paranoid thinking is associated with negative affect and mood and with worry (23).Our results seemed to suggest that neither negative affect nor workplace bullying mediate the association between paranoid thinking and the intentions to engage in negative behaviors in the workplace of French teachers. In other words, showing paranoid tendencies and feeling stressful and depressive in the workplace, when considered together, do not contribute to make French teachers engage in deviant behaviors. Nevertheless, individually they were both found to correlate with intentions to engage in negative behavior in the workplace. Again, these results suggested that the more French and UK teachers show paranoid thoughts that include thinking that others are conspiring against them and trying to harm them on purpose the more they show intentions to engage in retaliatory behaviors, regardless of the negative affect they experience in the situation.

Therefore, the mediation results of the French sample of teachers partially support the cognitive model for persecutory delusions proposed by Garety, Kuipers, Fowler, Freeman, and Bebbington (2) that proposed as observed in the French sample of teachers, that paranoia influences the presence of negative affect because paranoid thinking constitutes vulnerability to psychopathology, e.g., symptoms of depression, stress, and anxiety (23). However, and according to this model, depression, anxiety, and stress may also trigger and help to maintain paranoia because of the inherent depressive feelings of self-vulnerability and of self-depreciation that in their turn, may induce thoughts of possible threats posed by others against oneself (6, 23).

Under the light of Chan and McAllister's (1) model it is also proposed that the negative behaviors such as, workplace deviance are able to confirm and maintain the paranoid ideas of being subjected to a conspiracy and to mistreatment and abuse from others that are perceived to be undeserved. However, because factual workplace deviant behaviors were not measured, the results of the UK sample [against what was proposed by Chan and McAllister, (1)] only support a uni-directional relationship between paranoid cognitions, and cognitions related to the behaviors of workplace deviance and not actual deviant behavior. Indeed, it seems that paranoid cognitions are associated with intentions of workplace deviance and may feed into those intentions. This gives support to the notion that sub-clinical paranoia may be a stable trait predisposition that influences both cognitions and behavior (49, 50) in the workplace.

Previous research also found that ruminating on the paranoid thoughts might also cause more negative affect (6) and consequently this might lead to the onset of negative behavior in the workplace (e.g., deviant behavior) that has the purpose of managing unpleasant thoughts and feelings. Unfortunately, those negative behavioral strategies may help to maintain the paranoid ideas and associated negative affect in the long term (1). It is possible that eventually, a vicious circle may be set up with paranoia being associated with stress, anxiety and depression that in their turn may be associated with workplace deviance that in its turn may help to maintain the paranoia and associated negative affect/mood. This has yet to be tested in the literature in this area.

### Limitations and Future Research

There are several limitations with this study. Firstly, the sample sizes were rather small and most of the teachers were from the public sector and female. However, the samples of our study do represent national samples since for both UK and French national samples there are more females than males in the teaching profession. Nevertheless, it is suggested that future studies in this area should have larger sample sizes and should include more diversified samples of teachers, including teachers from the public and private sectors. Ideally, research should be done with more cross-cultural samples to provide a holistic picture to the issue of workplace deviance in teachers. Secondly, the samples were of convenience, which means that a self-selection bias might have been present. Thirdly, we used self-report measures of bullying and of workplace deviance. Although we conceptualized workplace bullying as perceptions of bullying and workplace deviance as intentions of deviance (i.e. cognitions and not actual behavior) it is argued that in the future and in contrast to most research that has been done up until now, studies should try to have observational measures for workplace bullying and deviance. Fourthly, clearly there were concerns of social desirability regarding the report of workplace-related issues and thoughts by the French teachers. Indeed, by using self-report questionnaires to measure those issues and thoughts, not only they fail to fully and vividly capture the experiences of bullying and associated paranoid thougths and workplace deviant behaviors but there might have been social desirability biases in the reporting of behaviors, bullying, negative affect/mood and of paranoia (51). As such, it is recommended that qualitative methods e.g., interviews or focus groups should be coupled with self-report questionnaires to fully capture the experiences and narratives of bullying reported by workers (52). Moreover, this study being cross-sectional cannot ascertain causality. This highlights the need of finding different ways of gaining data from French teachers also highlighting the need to use longitudinal designs for this area of research, for example, measuring the onset of paranoia and bullying through longitudinal assessments like weekly diaries of workplace issues.

Finally, there are potential confounders like career adaptability, affective investment, organizational and peer support that are directly related to the workplace environment and as such, might have influenced teachers' responses in this study. For example, career adaptability has been found to be essential for strategic career development, enabling adjusting to adverse work environments (53). Therefore, career adaptability may influence or not the onset of workplace deviant behaviors of teachers. Moreover, organizational support has been found to moderate the effect of workplace bullying on workers' workplace deviance and paranoia (8). Other studies also found support for the role of perceived organizational support in reducing emotional exhaustion and in improving personal accomplishment (two indicators of burn-out) in special education teachers (54, 55). Also, studies have found that workers' affective commitment on the one hand is positively related to workers' self-efficacy and career adaptability (56) and on the other hand, is negatively related to workers' deviant behaviors (57). Therefore, all of those variables should be studied in the future in relation to teachers' paranoia and workplace bullying and deviance.

### Practical Recommendations

The results of this study suggest important practical recommendations. A psycho-social intervention should be devised for teachers with the intent of promoting their positive mental health and positive behaviors in schools but this needs to be tailored according to the cultural context. This intervention should integrate components of cognitive and behavioral therapy (CBT) tailored to address workplace contexts/issues (7, 8). CBT might help teachers deal with the distress that may be caused by thoughts of paranoia and associated symptoms of depression, anxiety, and stress and by experiences of workplace bullying by teaching them problem solving skills, coping skills and skills in managing stress. CBT techniques may be employed to help teachers change the function and impact of negative thoughts (e.g., paranoia) and feelings (e.g., cognitive restructuring and cognitive reappraisal). Second, the intervention should incorporate components of teachers' professional training and development (58, 59) that address skills of career adaptability, conflict-resolution, self-efficacy and social-skills training. The intervention should also address workplace bullying in schools, how to identify it and report it and how to effectively manage bullying in schools and prevent it. School headmasters, teachers' colleagues and supervisors, institutional staff, parents, and students should also go through an intervention on bullying to be able to address this problem effectively and conflict resolution strategies should be taught to teachers, supervisors, headmasters, parents, and students and should be put into practice. Moreover, it is important to recognise in schools how a “toxic” environment that is characterized by teachers' paranoia and victimization may be behind their display of workplace deviance. Therefore, components of the psycho-social intervention for teachers should also address their workplace deviant behaviors and how they might negatively affect not only their relationships with staff, parents and students but the school environment, productivity and the students' achievement (55, 60) and well-being. Teachers should be taught on how to behave ethically by role modelling positive and ethical behavior, thus impacting positively on students' academic achievement and well-being.

### Conclusions

Our results showed that UK teachers tend to report more work-related bullying and intentions to engage in workplace deviance than French teachers. More importantly, results suggested that paranoid thinking not only is rather common in UK and French teachers but is also associated with perceptions of workplace bullying and intentions to engage in workplace deviance. Moreover, our results showed that neither negative affect nor workplace bullying mediate the association between paranoid thinking and intentions to engage in workplace deviance in both French and UK teachers. This supported previous research with UK workers that found that their supervisory-related paranoia had an impact on their poor well-being and intentions to engage in workplace deviance (8). Future studies should explore how other potential mediators factors, such as rumination might mediate the impact of paranoia on intentions of workplace deviance (6). Although our results only partially support the cognitive models of paranoia (2), it is essential to further investigate how the clinical literature can contribute to the understanding of critical problems that are mostly described in organizational literature such as, workplace bullying and deviance, investigating as well how these might contribute to the onset of mental health issues. Hence, results supported the usefulness of marrying organizational and clinical literature and showed that cognitive and behavioral classical models of paranoia (2, 23) can be applied to the workplace to understand the relationships between paranoid thinking, workplace bullying, negative affect, and intentions of workplace deviance in teachers.

## Data Availability Statement

The datasets generated for this study are available on request to the corresponding author.

## Ethics Statement

The studies involving human participants were reviewed and approved by the Department of Psychology at Nottingham Ethics Research Committee and Laboratoire Epsylon Ethics research Committee in France. The patients/participants provided their written informed consent to participate in this study. The study was conducted under the guidelines of the British Psychological Society for internet mediated research, see here: https://www.bps.org.uk/news-and-policy/ethics-guidelines-internet-mediated-research-2017 and the code of ethics of the Declaration of Helsinki as it can be read in text under Participants.

## Author Contributions

All authors were involved in setting up the research. BL was involved in setting up the research in the UK whereas CB, SR, and VM were involved in setting up the research in France. BL was in charge of formulating the research questions and writing pieces of the introduction, method, results for the UK sample and the general discussion. CB was in charge of writing pieces of the introduction, the method, and the results for the French sample. SR and VM were in charge of writing pieces of the introduction for the French sample.

## Conflict of Interest

The authors declare that the research was conducted in the absence of any commercial or financial relationships that could be construed as a potential conflict of interest.

The handling editor declared a past collaboration with one of the authors SR.
